# Biomimetic on-chip assay reveals the anti-metastatic potential of a novel thienopyrimidine compound in triple-negative breast cancer cell lines

**DOI:** 10.3389/fbioe.2023.1227119

**Published:** 2023-09-28

**Authors:** Indira Sigdel, Awurama Ofori-Kwafo, Robert J. Heizelman, Andrea Nestor-Kalinoski, Balabhaskar Prabhakarpandian, Amit K. Tiwari, Yuan Tang

**Affiliations:** ^1^ Biofluidics Laboratory, Department of Bioengineering, College of Engineering, University of Toledo, Toledo, OH, United States; ^2^ Department of Biomedical Engineering, College of Engineering, University of Michigan, Ann Arbor, MI, United States; ^3^ Department of Surgery, College of Medicine and Life Sciences, University of Toledo, Toledo, OH, United States; ^4^ Biomedical Technology, CFD Research Corporation, Huntsville, AL, United States; ^5^ Department of Pharmaceutical Sciences, College of Pharmacy, University of Arkansas for Medical Sciences, Little Rock, AR, United States

**Keywords:** endothelial cell, intravasation, microfluidics, permeability, tumor microenvironment

## Abstract

**Introduction:** This study presents a microfluidic tumor microenvironment (TME) model for evaluating the anti-metastatic efficacy of a novel thienopyrimidines analog with anti-cancer properties utilizing an existing commercial platform. The microfluidic device consists of a tissue compartment flanked by vascular channels, allowing for the co-culture of multiple cell types and providing a wide range of culturing conditions in one device.

**Methods:** Human metastatic, drug-resistant triple-negative breast cancer (TNBC) cells (SUM159PTX) and primary human umbilical vein endothelial cells (HUVEC) were used to model the TME. A dynamic perfusion scheme was employed to facilitate EC physiological function and lumen formation.

**Results:** The measured permeability of the EC barrier was comparable to observed microvessels permeability *in vivo*. The TNBC cells formed a 3D tumor, and co-culture with HUVEC negatively impacted EC barrier integrity. The microfluidic TME was then used to model the intravenous route of drug delivery. Paclitaxel (PTX) and a novel non-apoptotic agent TPH104c were introduced via the vascular channels and successfully reached the TNBC tumor, resulting in both time and concentration-dependent tumor growth inhibition. PTX treatment significantly reduced EC barrier integrity, highlighting the adverse effects of PTX on vascular ECs. TPH104c preserved EC barrier integrity and prevented TNBC intravasation.

**Discussion:** In conclusion, this study demonstrates the potential of microfluidics for studying complex biological processes in a controlled environment and evaluating the efficacy and toxicity of chemotherapeutic agents in more physiologically relevant conditions. This model can be a valuable tool for screening potential anticancer drugs and developing personalized cancer treatment strategies.

## Introduction

Breast cancer (BC) is the second leading cause of cancer deaths in women and the most common cancer in the United States (American Cancer Society, 2022). BC accounts for about 30% of new female cancer cases each year, and about 1 out of 8 women will develop BC at least once in their lifetime (Centers for Disease Control and Prevention, 2021). With immunohistochemistry (IHC), BC is classified by the presence/absence of Estrogen Receptor (ER), Progesterone Receptor (PR), and Human Epidermal Growth Factor Receptor 2 (HER2/neu) (Tang et al., 2016). Hormone^+^ or HER2^+^ BC subtypes respond to targeted therapies. However, triple-negative breast cancer, the BC subtype lacking ER, PR, or HER2 expression, presents the biggest obstacle in BC treatment. TNBC accounts for 15% of the 281,550 new invasive BC cases diagnosed annually (Dietze et al., 2015; Garrido-Castro et al., 2019), while it is responsible for 25% of all BC deaths (Howlader et al., 2018).

Due to a lack of effective targeting therapies (Tang et al., 2016), treatment of TNBC heavily relies on conventional chemotherapy, such as Taxanes (paclitaxel, docetaxel, cabazitaxel, and abraxane) as the standard of care (Hudis and Gianni, 2011; Park et al., 2018). Taxanes, such as paclitaxel, produce their efficacy by targeting and stabilizing microtubules during the G2-M phase of the cell cycle, thereby inhibiting the depolymerization of microtubules into soluble tubulin (Malla et al., 2022b). The consequent cell cycle arrest at the mitotic phase eventually leads to apoptotic cell death (Wang et al., 2000). Most TNBC patients respond to treatment initially; however, resistance eventually develops (Kalimutho et al., 2015), resulting in cancer recurrence and a worse prognosis (Gandhi et al., 2015). This is because TNBC cells can acquire mutations in tumor suppressor genes (e.g., *PTEN*, *p53*) and develop resistance to apoptosis (Stephens et al., 2012). TNBC cells can also develop resistance by increasing DNA repair or overexpressing efflux transporters (Rodriguez et al., 2010; Tiwari et al., 2011).

Studies suggest apoptosis-inducing chemotherapeutics such as PTX can cause changes in the primary tumor microenvironment that favor metastasis (Daenen et al., 2011; Vyas et al., 2014; Harney et al., 2015; Karagiannis et al., 2017a; Karagiannis et al., 2017b; Chang et al., 2017; Middleton et al., 2018; Karagiannis et al., 2019; Keklikoglou et al., 2019). Most conventional chemotherapeutics act as a “stressor,” resulting in tissue hypoxia and apoptosis in the primary tumor microenvironment, which in turn activates the “stress-reading” cells, such as tissue-resident macrophages, fibroblast, and ECs (Karagiannis et al., 2018), leading to the release of proinflammatory mediators (cytokines and chemokines) locally and systemically (Balkwill and Mantovani, 2001; Mantovani et al., 2008; Grivennikov et al., 2010; Galdiero et al., 2018). Locally, inflammation reeducates TNBC cells in the primary tumor, enhancing their intravasation. Systemically, the released cytokines and chemokines facilitate premetastatic niche formation, which induces endothelial activation and microvascular hyperpermeability (Huang et al., 2009), enhancing the extravasation (Peinado et al., 2017). PTX also displays an elevated cytostatic and cytotoxic effect on vascular ECs due to microtubule disruption (Pasquier et al., 2004; Pasquier et al., 2005; Gallego-Jara et al., 2020; Mosca et al., 2021) and oxidative stress (Pasquier et al., 2004), among others. These effects of PTX inhibit normal endothelial angiogenesis (Pasquier et al., 2004; Pasquier et al., 2005; Bocci et al., 2013), thus limiting its therapeutic potential in patients (Pasquier et al., 2004; Pasquier et al., 2005; Bocci et al., 2013).

In summary, treating TNBC is particularly challenging due to drug resistance and increased DNA repair (Rodriguez et al., 2010), the development of genetic mutations that decrease the likelihood of apoptosis (Radetzki et al., 2002; Kalimutho et al., 2015), and exacerbated metastasis due to apoptosis-inducing chemotherapy (Ricci and Zong, 2006; Karagiannis et al., 2018). Novel chemotherapeutics which functions through an apoptosis-independent cancer cell-killing mechanism are urgently needed to treat drug-resistant, metastatic TNBC. Our team has discovered a new class of thieno-pyrimidin-4-yl-hydrazinylidene compounds (TPH analogs) and has recently reported a novel thienopyrimidine analog TPH104 that can selectively induce necroptotic cell death in TNBC cells (Tukaramrao et al., 2021). Prompted by the interesting anti-TNBC efficacy of TPH104, we designed and evaluated structurally related analogs that yielded a lead compound, ***TPH104c***, which had potent anti-TNBC activity and induced necroptotic cell death in TNBC cells in *in vitro* well plate experiments.

We engineered a vascularized microfluidic model of the TNBC tumor microenvironment using a commercially available microfluidic platform. This model allowed us to assess the anti-TNBC efficacy of the novel compound TPH104c, as well as its potential cytotoxic effects on vascular ECs. In this model, drug-resistant, metastatic human TNBC cells were successfully co-cultured alongside primary human ECs in distinct compartments connected through a microfabricated porous structure. Concurrently, TPH104c and PTX were perfused through the vascular channels, closely mimicking the systemic drug delivery route *in vivo*. Utilizing this model, we demonstrate the distinct selectivity of TPH104c towards TNBC cells in contrast to ECs. This is evident in its higher cytotoxicity and anti-metastatic effect on cancer cells, coupled with its lower impact on endothelial barrier integrity. Overall, this model presents an innovative approach to anti-metastatic efficacy testing by incorporating co-cultured human TNBC cells and primary ECs within an optically clear device, reproducing tumor perfusion and allowing for real-time assessment of therapeutic responses and interactions between the tumor and ECs, thus addressing a critical need for high-fidelity *in vitro* testing of anticancer therapeutics.

## Materials and methods

### Cell culture, reagents, and microfluidics

Human Umbilical Vein Endothelial Cells (HUVECs, CC-2519) were obtained from Lonza (Basel, Switzerland); Endothelial Cell Growth Medium (EGM; PM211500) was purchased from Genlantis (San Diego, California). Dulbecco’s Modified Eagle Medium (DMEM, 10-013-CV), Fetal Bovine Serum (FBS, 35-016-CV), and Penicillin-Streptomycin (Pen-Strep, 15140-148), Human Plasma Fibronectin (341635), Matrigel (47743-722), CellTiter-Blue Reagent (G808A), Phosphate Buffered Saline (PBS; SH30356.01), 75 cm^2^ Culture flasks (130190), 4 kDa Texas Red Dextran (T1037-100MG), PTX (Molecular Weight: 853 Da) (T1912-5MG), 0.25% Trypsin-EDTA (25200-056), Anti-Hu CD144 (VE-Cadherin; 14-1449-82), Goat anti-Mouse IgG (H+L) Highly Cross-Adsorbed Secondary Antibody Alexa Fluor Plus 488 (A32723), Fixative solution (FB002), Triton X-100 (648463-50ML), Normal Goat Serum (31872), NucBlue Live Cell Stain Ready Probes (R37605), SlowFade Gold antifade reagent (S36936), Dulbecco’s Phosphate Buffered Saline/Modified (DPBS 1X; +Calcium, +Magnesium; SH30264.01), CellTracker CM-DiI (C7001), CellTracker Green CMFDA (C7025), Dimethyl Sulfoxide (DMSO, CAS 67-68-5), 3-(4,5-Dimethylthiazol-2-yl)-2,5-diphenyltetrazolium bromide (MTT) (M6494), and Isopropanol, 70% v/v (A459-4) were purchased from Fisher Scientific (Hampton, New Hampshire).

**FIGURE 1:**
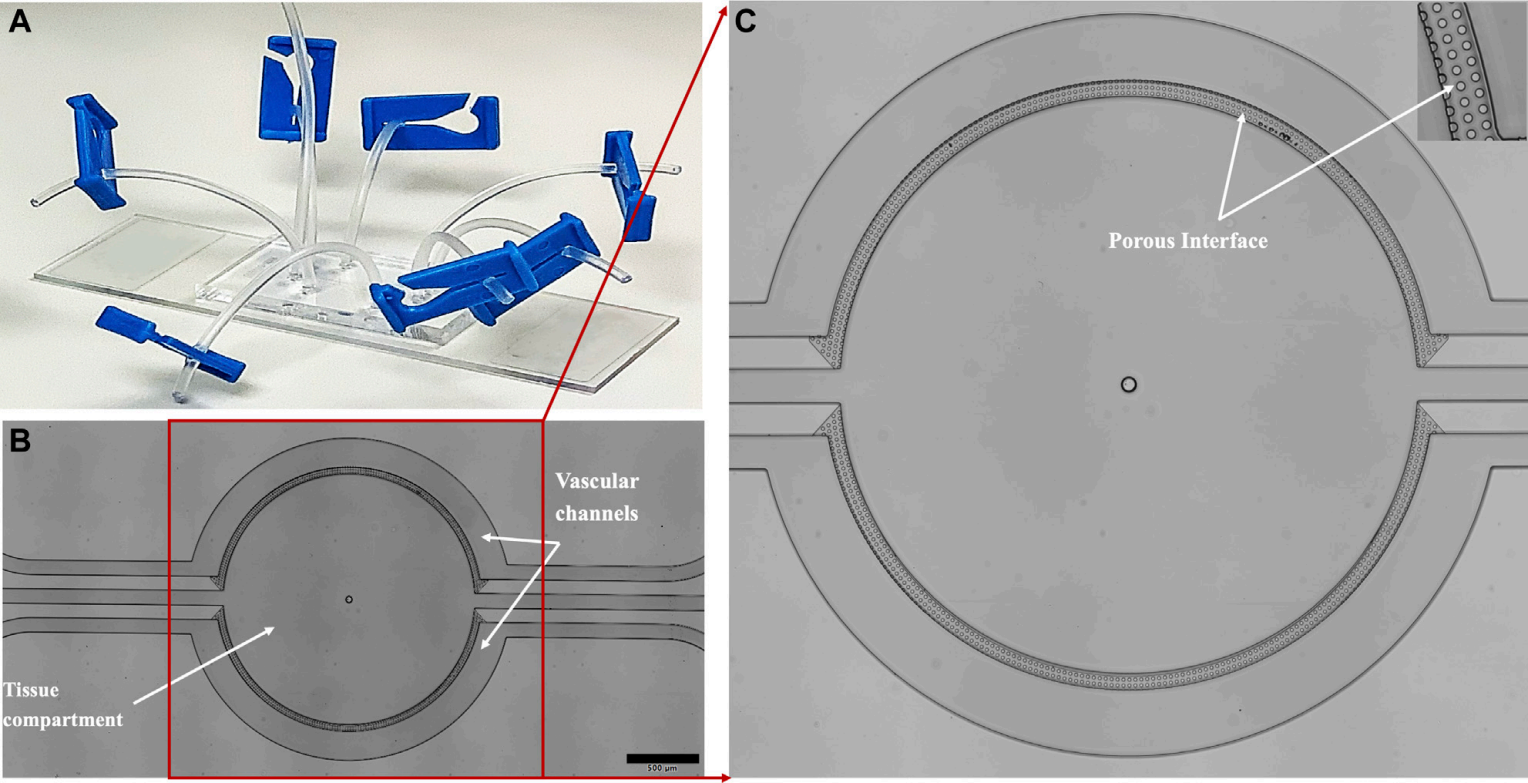
The **(A)** microfluidic device is fabricated in transparent PDMS and bonded to a glass slide. The device comprises **(B)** two semicircle vascular channels (basolateral side) surrounding a tissue compartment (apical side), all of which are individually perfused via inlet/outlet ports connected with Tygon tubing. The vascular channels and the tissue compartment are separated by a **(C)** porous interface comprising of microfabricated porous structure, which facilitates tumor-endothelial co-culture and information/mass exchange.

The TPH104c (Molecular Weight: 390.47 Da) compound was synthesized in Dr. Tiwari’s laboratory, and SUM159PTX (TNBC) cell line was developed in collaboration with Dr. Tiwari’s lab (Sridharan et al., 2019). SUM159PTX (parent cell line SUM159PT) is a highly metastatic, drug-resistant triple-negative breast cancer cell line. The microfluidic devices (Figures 1A–C) were purchased from SynVivo Inc. (SynTumor 102012, Huntsville, Alabama). HUVECs were cultured with EGM, and DMEM supplemented with 10% FBS and 1% Pen-Strep were used to culture cancer cells. All cells were grown in tissue culture flasks inside a 37°C incubator with 5% CO_2_ and 95% humidity. Cells were sub-cultured when they reached 80%-90% confluency. HUVECs were used between Passages one to five, whereas SUM159PTX was utilized until passage 20.

### Establishing tumor-EC co-culture in the microfluidic device

The microfluidic device (SynTumor 102012, Figures 1A–C) was used to create the tumor microenvironment. The device constructed using soft lithography technique (Tang et al., 2017; Soroush et al., 2018; Soroush et al., 2019), comprises a polydimethylsiloxane (PDMS) based, disposable, and optically clear microfluidic chip that contains a tissue compartment for the culture of breast cancer cells and ECs cultured in the surrounding vascular channels. The two compartments communicate via microfabricated 8 μ m porous structures. The microfluidic device is bonded to a microscope slide for easy imaging and real-time monitoring of the cells.

The ECs were seeded in the two vascular channels with flow perfusion, whereas the tissue compartment was seeded with drug-resistant TNBC embedded in Matrigel for tumor formation in 3D. There is continuous media perfusion through the vascular channels in this platform, mimicking the blood flow and the exchange of nutrients, oxygen, and metabolites. The continuous medium perfusion was generated by connecting the device to an external automated syringe pump (PHD ULTRA, Harvard Apparatus, 70-3007). The induced perfusion shear stress was about 0–1.75 dyne/cm^2^, sufficient to reflect the physiological blood flow in the microvasculature.

**FIGURE 2:**
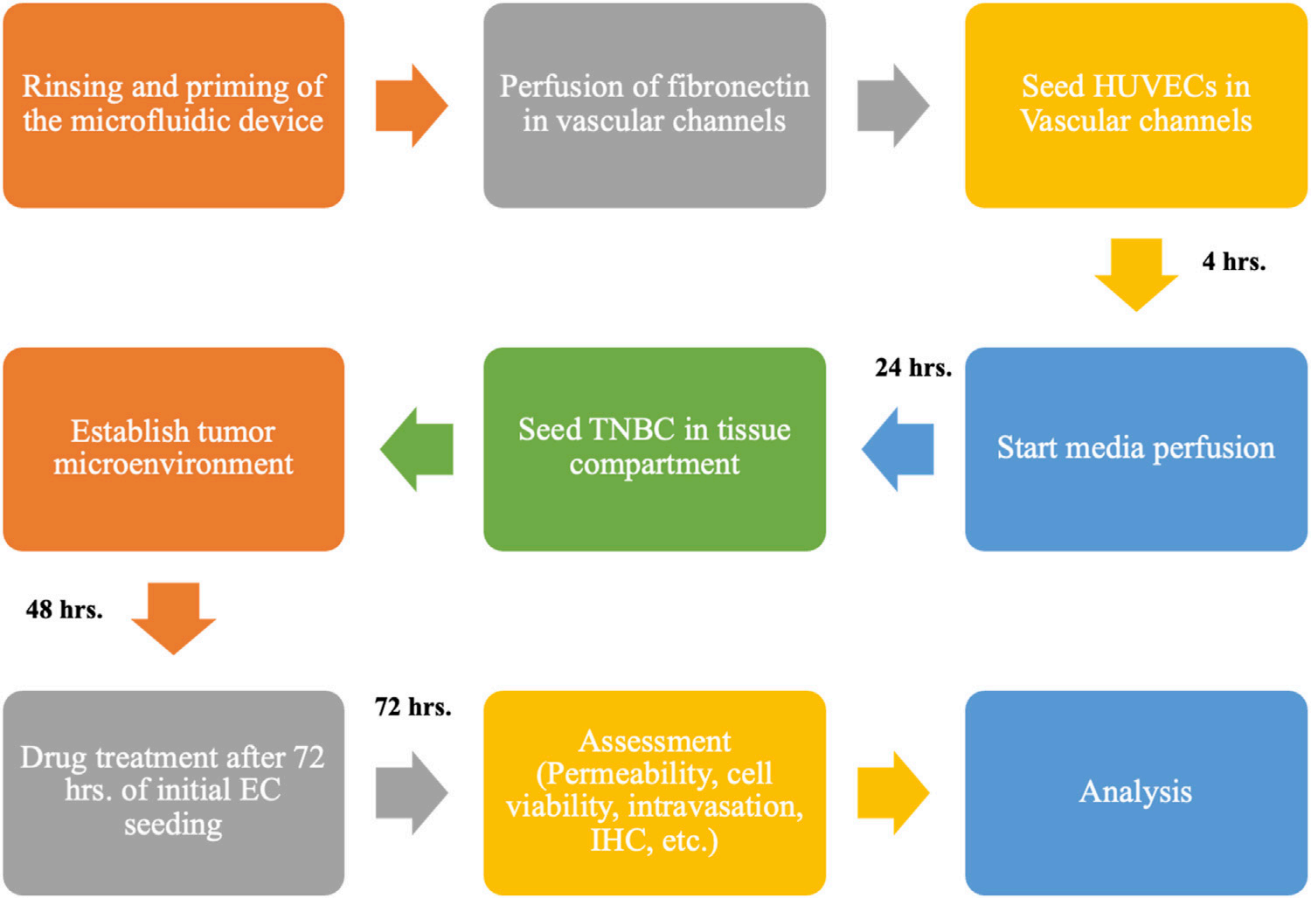
Flowchart shows the overall experimental setup for creating the HUVEC-TNBC co-culture model under flow perfusion and the timeline for using the biomimetic 3D tumor model to test the anti-TNBC efficacy of chemotherapeutics, TPH104c, and PTX using various biological assays.

A flowchart detailing the creation of the model is illustrated in Figure 2. To establish the co-culture, the microfluidic devices were rinsed with deionized (DI) water and degassed using a pneumatic primer (N_2_ gas at 7 psi for 15 min) to eliminate air bubbles. After degassing, the tissue compartment was pre-treated with 5% Matrigel solution (in serum-free DMEM) followed by 20 min incubation at 37°C. The devices were then perfused with human fibronectin (200 μg/mL in PBS) and incubated for 1 hour at 37°C to produce a homogenous coating to promote cell attachment. All devices were rinsed with fresh EGM before cell seeding.

HUVECs (3.5 × 10^7^ cells/mL), suspended in EGM supplemented with 20% FBS, were introduced into the vascular channels via a precision syringe pump (Pump 11 Elite, Harvard Apparatus) at a 5 μL/min flow rate. The exact process was repeated to seed HUVECs in the other vascular channel. The microfluidic devices were placed in the cell culture incubator for 4 hr to facilitate cell attachment. Afterward, the vascular channels were manually rinsed with EGM to flush out debris and unattached cells. After initial seeding, the HUVECs were subjected to continuous media perfusion to mimic the *in vivo* flow shear conditions. The perfusion level in both vascular channels is controlled simultaneously by a precision syringe pump (PHD ULTRA, Harvard Apparatus) with a multirack attachment. Tygon Microbore tubing with an outside diameter of 0.06 inch (1.524 mm) and inner diameter of 0.02 inch (0.508 mm) served as the connecting ports for the fluidic and pneumatic interface. A stepwise linearly increased shear stress scheme was applied to facilitate EC physiological function and lumen formation (no shear, 4 h; expansion, 0–1.75 dyne/cm^2^, 24 h; quiescent culture, 1.75 dyne/cm^2^ maintained till the conclusion of the experiment).

Briefly, TNBC cells (10^7^ cells/mL) resuspended in 5% Matrigel/serum-free media was introduced into the tissue compartment using a precision syringe pump (Pump 11 Elite, Harvard Apparatus). The TNBC cells were seeded 24 h after HUVEC seeding using our established methods (Yang et al., 2021). The devices were then kept inside incubator for another 48 h for the tumor to develop, after which the device was ready for further testing described below. The cancer cells in the tissue compartment were perfused via bolus injection (10 min media perfusion at 1 μ L/min) every 8 h throughout the experiment.

### Image acquisition

Fluorescence and bright field images were acquired using an Olympus IX71 inverted microscope equipped with a Proscan XYZ 3D automated stage and a Hamamatsu ORCA Flash 4 camera. Olympus cellSens Dimension with Multi-Position Solutions software package and NIH ImageJ software were used to post-process captured images and videos. Each device was imaged daily using the stitch image feature of the CellSens software at 10X or ×4 magnification up to the point of the experiment. The detailed image acquisition procedure for each experiment is described below. This microscope system was used for on-chip drug efficacy study including permeability measurement (Figure 7), cell viability and proliferation assay (Figures 5, 6), intravasation assay (Figure 8) as well as immunofluorescence staining of VE-Cadherin and phalloidin in ECs (Supplementary Figure S1).

Confocal images were acquired using Leica TCS SP5 laser scanning confocal microscope with a 20X dry 0.7NA (Numerical Aperture) objective. Images were acquired in the XYZ plane with a tile scan of 512 × 512 pixels. Imaging was done sequentially with excitation and emission of Alexa fluor 488 and CM-DiI 553 at 488_ex_/518_em_ and 488_ex_/570_em_ and DAPI at 400_ex_/460_em_ in 3 μm steps. Images represent the projections of z stacks in xyzs mode. This microscope system was used to image the entire device with cells in different z planes (Figure 4).

### On-chip drug efficacy study

The efficacy of TPH104c against drug-resistant TNBC cells was tested in the microfluidic device and compared to the most common FDA-approved chemotherapeutic for TNBC cell treatment, PTX. TPH104c (0.1, 1, 10 µM in EGM) or PTX (0.1, 1, 10 µM in EGM) were introduced into the vascular channels using a precision syringe pump (PHD Ultra with a multirack attachment, Harvard Apparatus) to mimic the intravenous route of drug delivery *in vivo*. The vascular channels were perfused with a drug solution for 72 h where each device was imaged daily. After 72 h of drug treatment, permeability assay and cytotoxicity assays were performed to quantify the effects of drug treatments on HUVEC barrier integrity and cytotoxicity in both cells.

### Permeability measurements

To characterize the effects of drug treatments on EC barrier integrity, the permeability of the endothelial barrier was quantified by measuring the diffusion of 4 kDa TRITC dextran from vascular channels to the tissue compartment after 72 h of drug treatment. Following our already published protocol (Tang et al., 2017), the vascular channel of the microfluidic device was connected to a Hamilton syringe (GASTIGHT 1001) filled with dye solution and driven by a programmable syringe pump (Pump 11 Elite, Harvard Apparatus). The microfluidic device was then mounted onto the automated stage of the Olympus microscope system (Olympus IX71). At the time of the experiment, the dye solution was prepared using EGM to get a final concentration of 0.25 mM. The dye solution was then set to perfuse in the vascular channel with a flow rate of 1 μL/min (1.75 dyn/cm^2^) for 1 h. In the meantime, time-lapse images were taken every 1 min over 1 h using cellSens Dimension software and the Olympus microscope system. Region of Interests (ROIs) were drawn (Supplementary Figure S2) and the obtained intensity values were analyzed, and the permeability was calculated with the following equation:

$$P=\left(1-H_{CT}\right)\;\left(\frac{1}{I_{0}}\right)\left(\frac{dI}{dt}\right)\left(\frac{V}{S}\right)$$

*H*_*CT*_ = hematocrits in the vascular channel. In our case, H_CT_ = 0 (EGM does not contain blood cells). *I* = average intensity in the tissue compartment at a given time point, *I*_*0*_ = maximum fluorescence intensity in the vascular channel, and *V/S* = volume to surface area ratio of the vascular channel (Tang et al., 2017; Tang et al., 2018; Soroush et al., 2020; Yang et al., 2021).

### Cell viability and proliferation

CellTiter-Blue Cell Viability Assay was performed to determine the drug efficacy on cell viability and proliferation on-chip according to the manufacturer’s instructions. Briefly, the device was perfused with CellTiter-Blue reagent at a ratio of 1:5 with EGM and incubated for 4 h at 37 °C after 72 h of drug treatment. After incubation, the device was first placed in an orbital shaker for 15 s, then mounted to the Olympus IX71 microscope for fluorescence imaging. Finally, the images were loaded into the cellSens Dimensions software, and the intensity values were obtained, correlating with cell viability. In addition, standard MTT assay were performed in HUVECs and TNBC cells to determine their viability following TPH104c or PTX treatment. The assay was carried out on 96-well plates as per the manufacturer’s instructions.

### Tumor intravasation

TNBC intravasation from the tissue compartment, across the EC barrier, into the vascular channels was monitored by fluorescence imaging. For this purpose, CellTracker CM-DiI dye, which is non-toxic, well retained, and allows for multigenerational tracking of cellular movements (Krishnamurthy et al., 2008), was used. Briefly, TNBC cells were stained with CM-DiI dye before they were introduced into the tissue compartment. Meanwhile, HUVECs were stained with CellTracker Green CMFDA dye for better visualization of TNBC intravasation. Fluorescence images were acquired using the Olympus IX71 microscope system, as explained above. Images were then analyzed in cellSens Dimension software for quantifying intravasation. This was achieved using the “Count and Measure” function in cellSens Dimension software, where ROIs were drawn in different areas of the image as shown in Supplementary Figure S3. The obtained cell numbers were then analyzed and quantified (Detailed steps are given in Supplementary Material).

### Immunofluorescence staining

The formation of endothelial cell-to-cell adherens junctions was characterized using immunostaining against VE-cadherin. The devices were gently perfused with PBS. Cells were fixed using 4% formaldehyde at 4 °C for 10 min, after which it was rinsed twice with PBS and permeabilized with 0.1% Triton X-100 for 10 min at 4 °C. The device was again rinsed twice with PBS, followed by blocking with 5% normal goat serum diluted in PBS for 1 h at 37 °C. Monoclonal primary antibodies against VE-Cadherin were applied. After overnight incubation at 4°C, the corresponding fluorophore-tagged goat anti-mouse secondary antibodies were applied to the device for 1 h at 37 °C. Finally, the device was rinsed, counterstained with NucBlue (Hoechst 33342), and imaged using the Olympus system with cellSens Dimension software (Supplementary Figure S1) or using the Leica confocal microscope system (Figure 4).

### Statistical analysis

Experiments were repeated in triplicates. Statistical analyses were conducted using GraphPad Prism software version 9.5.1. A factorial design was used to determine the effects of each treatment on each cell type. Results were reported as mean ± standard deviation (SD) and analyzed by Analysis of Variance (*ANOVA*) (Gallego-Jara et al.). Significance levels were set at α = 0.05.

## Results

### An on-chip vascularized human breast tumor microenvironment was successfully established

A vital goal of this study is to mimic the tumor microenvironment to allow rapid signaling communication between the ECs and tumor cells while keeping them in separate compartments due to their very different culturing requirements. For this reason, a microfabricated porous barrier architecture (Figures 1B, C) based microfluidic device was used. The porous architecture (Prabhakarpandian et al., 2015; Tang et al., 2017), provides the communication area between the tissue area and the vascular channels.

**FIGURE 3:**
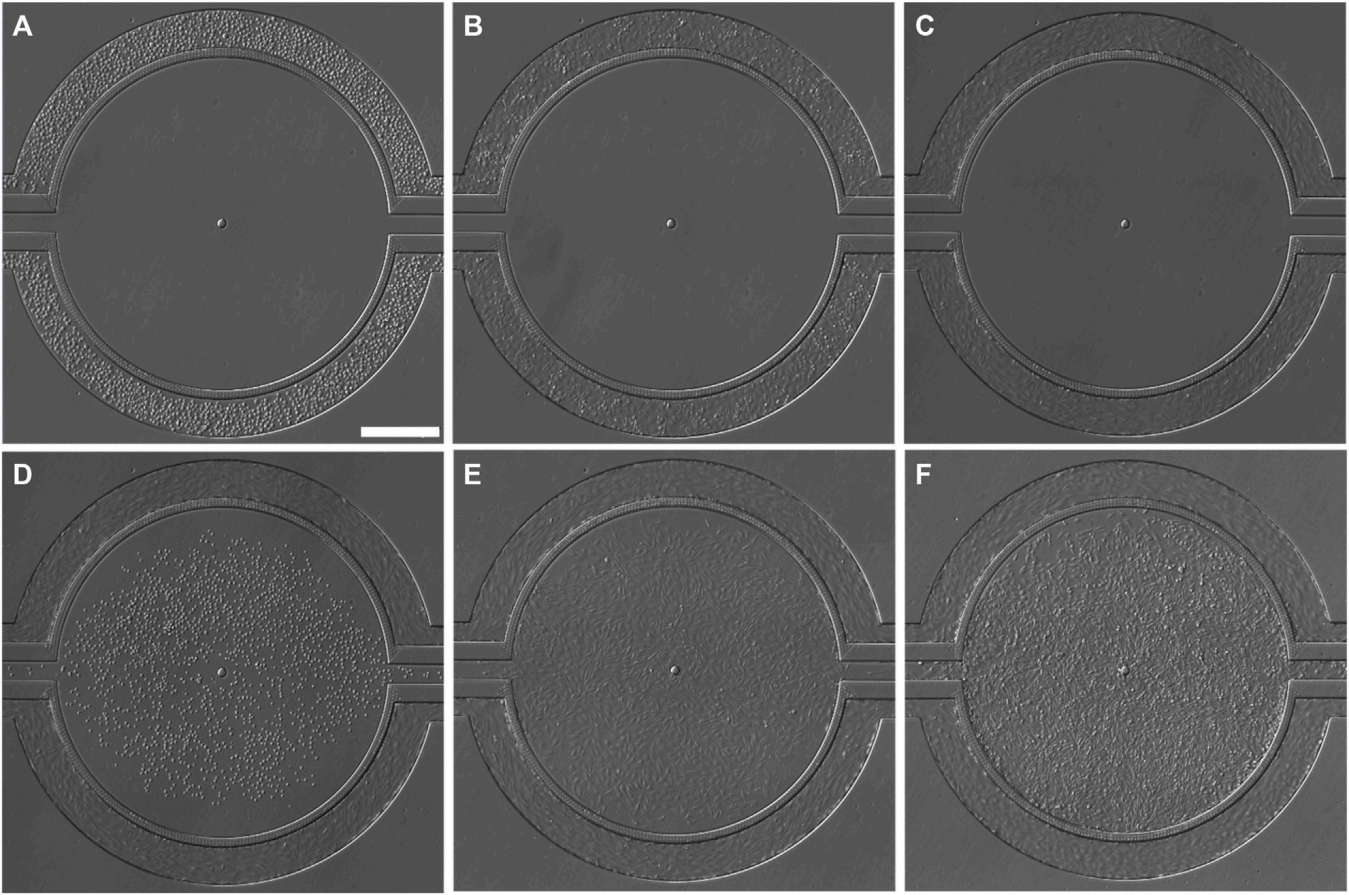
Brightfield images (BF) showing the progression for creating co-culture in the microfluidic device. **(A)** HUVECs, immediately after seeding, **(B)** HUVECs firmly attached to the fibronectin-coated vascular channel surface 4 h after initial seeding, no flow during this stage. **(C)** HUVECs aligned in the direction of flow in the vascular channels after 24 h of perfusion (0–1.75 dyne/cm2). **(D)** SUM159PTX cells, resuspended in 5% Matrigel, were seeded into the tissue compartment 24 h after HUVEC seeding. **(E)** SUM159PTX cells were allowed to attach and expand; 24 h after seeding. **(F)** The HUVEC-TNBC co-culture was fully established after 48 h after SUM159PTX seeding. Continuous media perfusion was maintained in the vascular channels, whereas bolus media injection was given every 8 h to the tissue compartment. Scale bar = 400 µm.

**FIGURE 4:**
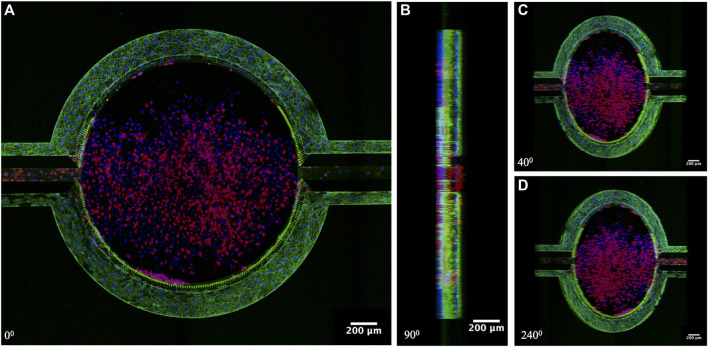
Confocal Images of the established tumor microenvironment on-chip. **(A)** Top view, **(B)** Side view (90°), **(C,D)** Angled view (40° and 240°). HUVECs formed a confluent monolayer in the vascular channels, whereas TNBC formed in the 3D tissue compartment. Red: TNBC cells (CM-DiI), Blue: Cell nucleus (Hoechst 33342), and Green: adherens junction between HUVECs (VE-Cadherin).

Co-culturing of tumor cells and ECs was achieved by first establishing ECs in the vascular compartment (Figures 3A–C), followed by culturing tumor cells in the tissue compartment (Figures 3D–F). Variable perfusion rates, as described previously, mimicking the blood flow pattern, were applied to vascular channels after HUVEC seeding to ensure dynamic shear stimulation of ECs to recapitulate the *in vivo* state. After 24 h of flow perfusion, HUVECs aligned along the direction of the fluid shear (Figures 3C–F; Supplementary Figure S1), which mimics the normal endothelium observed *in vivo* and is widely understood to be a defining morphological feature of the vascular homeostasis (Steward et al., 2015). After 72 h of flow perfusion, HUVECs formed a complete 3D lumen (Figure 4), covering all four sides of the vascular channels. This complete lumen is organized through strong intercellular contact as indicated by a prominent, continuous adherent junction signal via VE-cadherin staining (Figure 4; Supplementary Figure S1). VE-cadherin is vital for maintaining and controlling EC contacts, regulating vascular permeability, leukocyte extravasation, and various other cellular processes such as proliferation, apoptosis, growth factor receptor functions, and angiogenesis (Hynes, 1992; Kemler, 1992; Lampugnani et al., 1992; Gumbiner, 1996; Hendrix et al., 2001). Overall, these morphological features suggest the formation of a functional EC barrier under flow stimulation. Due to the unique side-by-side architecture, each cell type and its interactions were accessed simultaneously. After seeding (Figure 3D), TNBC cells quickly occupied the entire tissue compartment within 24 h (Figure 3E). Confocal imaging showed a homogeneous distribution of TNBC cells along the *z*-axis of the tissue compartment (Figures 4A–D **)**, suggesting 3D tumor formation.

### TPH104c exhibited selectivity to TNBC than endothelial cells

**FIGURE 5:**
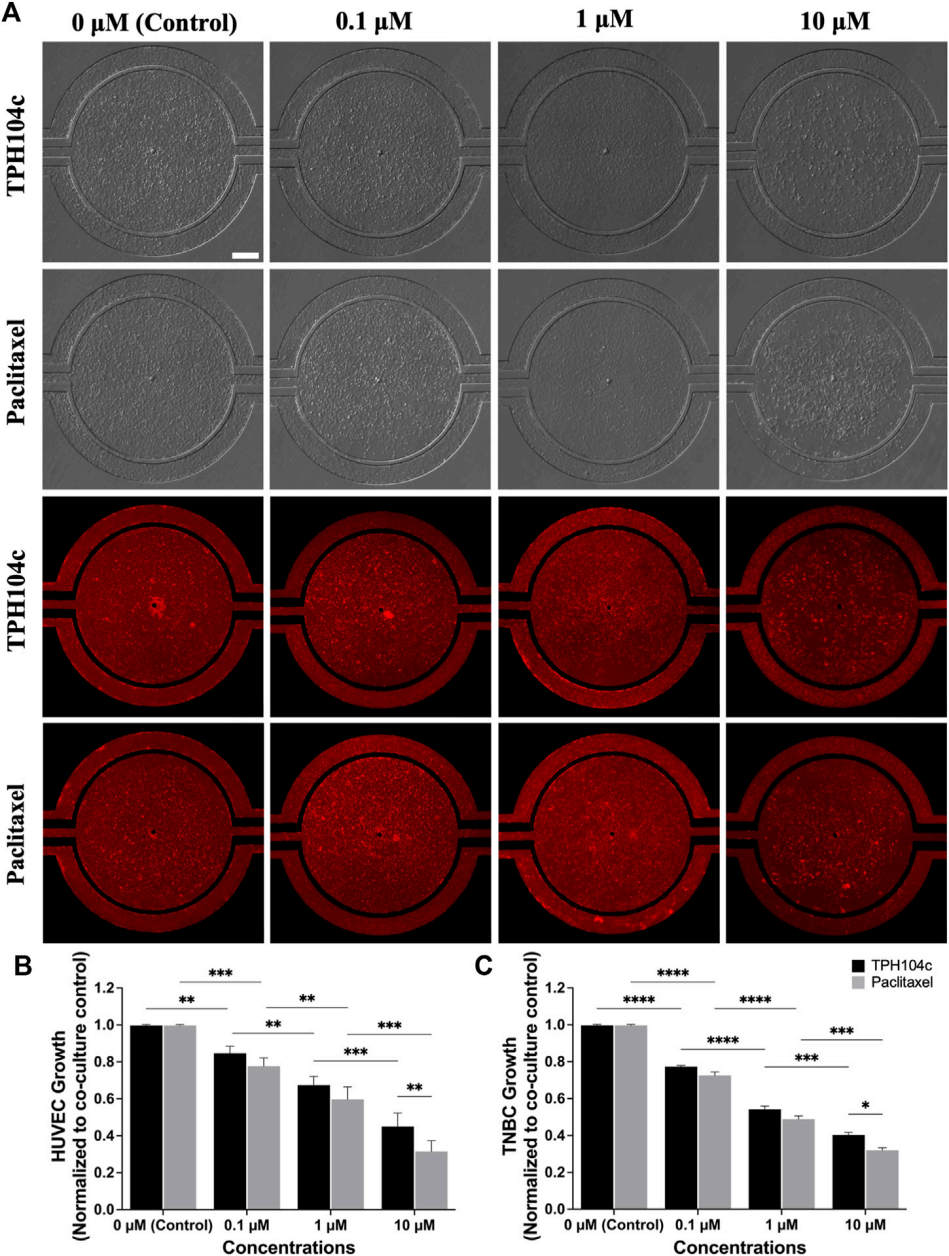
After 72 h of drug treatment, TPH104c exhibited superior selectivity to TNBC cells than ECs compared to PTX in the microfluidic TME, as indicated by **(A)** BF and fluorescence imaging after applying CellTiter-Blue cell viability assay **(B,C)** on HUVECs and TNBC cells. Data are presented as mean ± SD (*n* = 3). * *p* < 0.05, **p < 0.01, ***p < 0.001, ****p < 0.0001, by ANOVA. Scale bar = 200 µm.

A key feature of the tumor microenvironment model is its ability to mimic drug transport from the delivery site to the target tissue. In our microfluidic co-culture model, drug treatments successfully reached the tumor (central tissue compartment) from their initial delivery site (the vascular channels), resulting in concentration-dependent cancer cell killing and varying levels of EC damage (Figure 5). TPH104c treatment resulted in elongation and swelling in TNBC cells (Supplementary Figure S4). In contrast, cellular shrinkage and condensation after PTX treatment were observed (Supplementary Figure S4), which are typical hallmarks of apoptotic cell death (Elmore, 2007). This observation follows our previous findings, which suggest an necroptotic cell death induced by TPH104 (Tukaramrao et al., 2021), the parent compound of TPH104c.

Image analysis suggests significantly less EC damage by TPH104c treatment than PTX. Morphologically, the endothelial monolayer was largely intact after THP104c treatment at all concentrations tested, whereas, after PTX treatment, empty spots in the endothelial monolayer appeared at 0.1 µM concentration (Figure 5A). This loss of endothelial monolayer after PTX treatment was even more prominent at higher concentration treatments, where the endothelial monolayer became intermittent (1 µM) and completely disconnected (10 µM).

Quantitative cell viability assessment using the CellTiter-Blue assay revealed a similar trend (Figure 5B). TPH104c treatment at 0.1, 1, and 10 µM concentrations resulted in 15%, 33%, and 55% less HUVEC viability than control (co-culture, no drug treatment), respectively. In contrast, PTX treatments resulted in 22%, 40%, and 69% less HUVEC viability than the control. These findings suggest that TPH104c could be more favorable for intravenous drug delivery compared to PTX, owing to its capacity to cause minimal damage to the EC barrier. In contrast to the outcomes derived from the microfluidic model, MTT assays performed in HUVECs in well plate culture reveal a distinct disparity in the dose-response curves (Supplementary Figure S5). While PTX exhibited notably higher toxicity than TPH104c at lower concentrations (IC_50_ values of 0.075 and 5.34 µM respectively), there is no discernible difference in efficacy between the two treatments at higher concentrations.

In TNBC cells, both treatments exhibited a concentration-dependent drug action in the microfluidic model, with PTX exhibiting slightly higher cytotoxicity in TNBC cells (Figure 5C). MTT assays conducted in well plate culture indicate comparable cytotoxicity levels between PTX and TPH104c, with IC_50_ values of 5.12 µM and 7.41 µM respectively (Supplementary Figure S6). This consistency in response corresponds with our findings from the microfluidic platform (Figure 5C).

**FIGURE 6:**
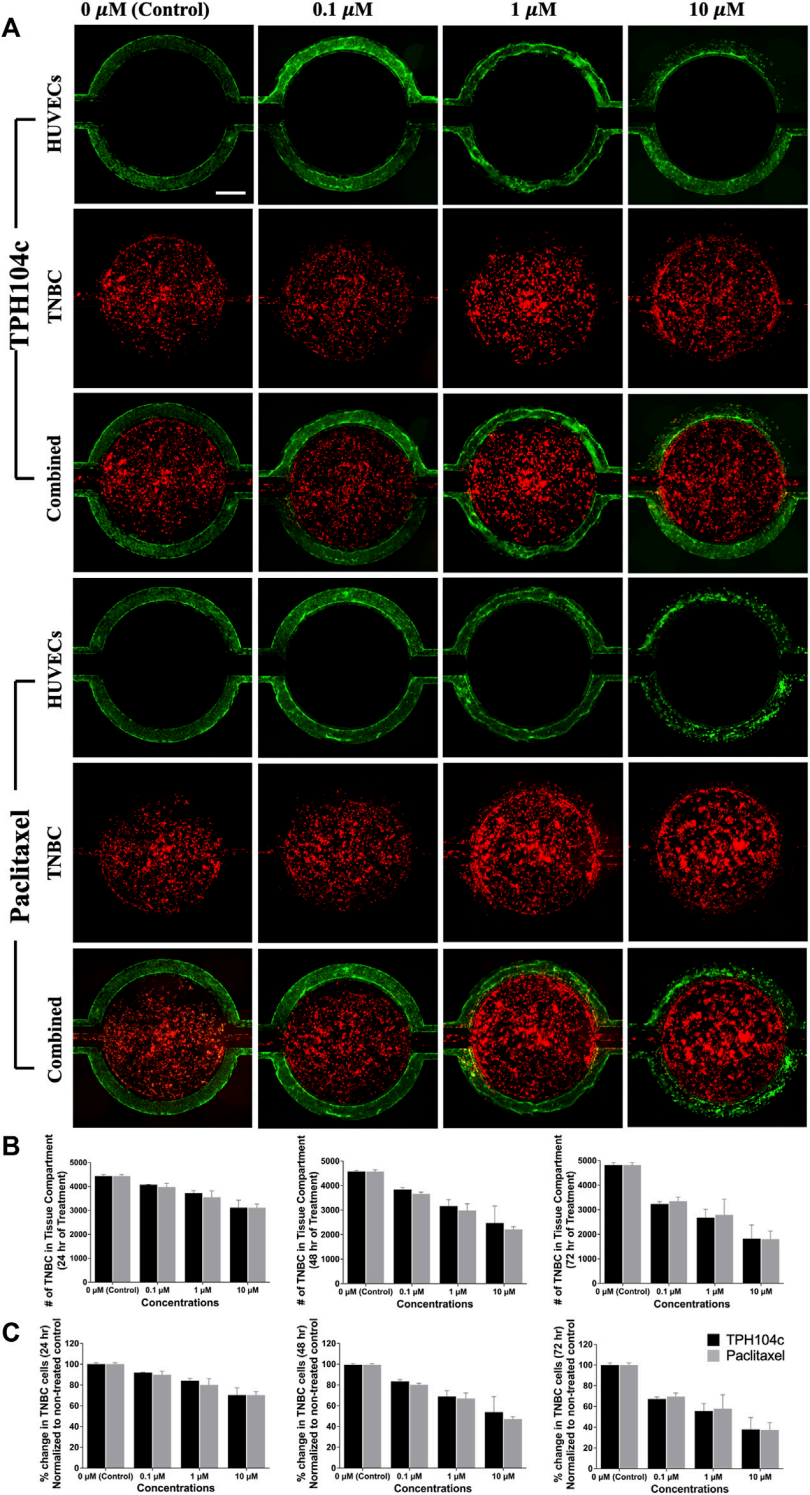
Drug treatments successfully reached the tumor (central tissue compartment) from their initial delivery site (the vascular channels). Images were taken at various time points during the 72-h drug treatment period, and the cell numbers were analyzed using the “Count and Measure” feature in cellSens Dimension software using the CM-DiI fluorescence signal. **(A)** Representative fluorescence images showing HUVECs (green), TNBC cells (red), and combined images with various concentrations of drug treatments. **(B)** Quantification of the number of surviving cells suggests concentration as well as time-dependent cancer cell killing for both treatments. **(C)** No significant efficacy difference was detected between the two treatments. Data are presented as mean ± SD (*n* = 3). Red: TNBC cells (CM-DiI) and Green: HUVECs (CMFDA). Scale bar = 400 µm.

Due to the fast permeation of CellTiter-Blue dye from the tissue compartment to the vascular channels, anti-TNBC efficacy assessment becomes challenging (as the dye enters the vascular channels and stains ECs, the number of ECs present can affect the final reading. In this case, since more ECs survived treatment with TPH104c compared to PTX, the TPH104c treatment group may produce a more false-positive signal due to the increased number of stained ECs). To solve this problem, a secondary fluorescence-based cell assay was performed. TNBC cells were stained with CM-DiI (Red) while HUVECs were stained with CMFDA (green) (Figure 6A). The efficacy of both drug treatments was then directly assessed by quantifying the number of surviving cells in the tissue compartment using CM-DiI fluorescence. As shown in Figure 6, both treatments exhibited concentration and time-dependent drug action. Meanwhile, no significant efficacy difference was detected between the two treatments (Figures 6B, C). Overall, TPH104c is equally potent in killing TNBC cells (Figures 5, 6) and, at the same time less toxic to ECs (Figure 5).

### Endothelial barrier function was largely preserved after TPH104c treatment

**FIGURE 7:**
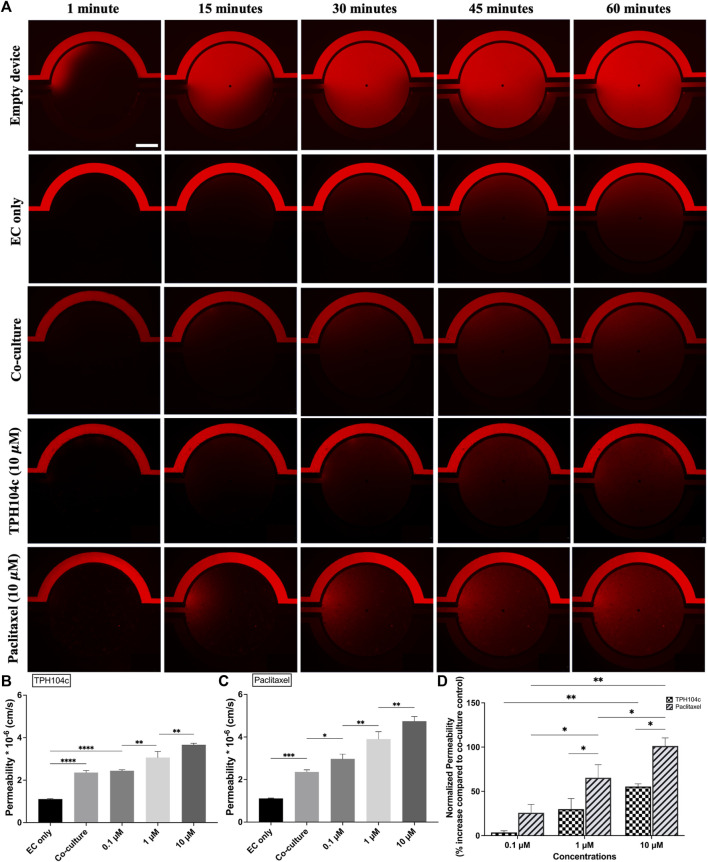
Endothelial barrier integrity was better preserved after the TPH104c treatment than PTX, as indicated by the permeability assay. The permeability of 4 kDa fluorescent dextran from the vascular channels to the tissue compartment was determined by measuring the fluorescent intensity of dextran in the tissue compartment relative to the vascular channel as the dextran diffuses from the vascular channel to the tissue compartment. **(A)** Representative fluorescence images of 10 μ M treatment groups compared to empty device, EC only control, and co-culture control at various time points. Increased permeability after **(B)** TPH104c or **(C)** PTX treatments suggest concentration-dependent damage to EC barrier integrity. Notably, **(D)** this increase was much more prominent in PTX treated than TPH104c treated groups. Data are presented as mean ± SD (*n* = 3). *p < 0.05, **p < 0.01, ***p < 0.001, ****p < 0.0001 by *ANOVA*. Scale bar = 400 µm.

Endothelial barrier function is important in maintaining the proper functioning of the circulatory system and preventing the infiltration of harmful substances into tissues (Mehta and Malik, 2006; Stan, 2007). It also plays a critical role in preventing cancer metastasis (Chen et al., 2013; Wettschureck et al., 2019; Liang et al., 2021). EC barrier function is maintained by cellular junctions between adjacent ECs, which limit the movement of substances through the intercellular spaces (Dejana et al., 2008). The barrier’s permeability can be altered by various factors, such as inflammation, injury, or disease, leading to the breakdown of the barrier and the leakage of fluids and substances into surrounding tissues (Mehta and Malik, 2006; Lampugnani, 2010). Permeability assays (Figure 7) were performed using 4 kDa dextran dye in EGM media and perfused for 1 h at 1 μ L/min flow rate (1.75 dyne/cm^2^), taking a time-lapse video every min. We measured permeability values of (2.07 ± 0.1042) × 10^–5^ cm/s in the empty device (no cells), (1.05 ± 0.0195) × 10^–6^ cm/s in the EC only control group (no TNBC, no drug treatment), and (2.3567 ± 0.1002) × 10^–6^ cm/s in the co-culture control (no drug treatment). This value is similar to reported *in vivo* values (Yuan et al., 2009; Shi et al., 2014) and is tighter than other reported *in vitro* models (Kazakoff et al., 1995; Lee et al., 2014; Frost et al., 2019).

Compared to co-culture control, a concentration-dependent permeability increase was observed after PTX or TPH104c treatment (Figures 7B, C). However, the endothelial barrier integrity was better preserved after the TPH104c treatment than PTX. The permeability values after 0.1, 1, or 10 μ M TPH104c treatment increased by 4%, 30%, and 55%, respectively, compared to co-culture control. However, the permeability values after 0.1, 1, or 10 μ M PTX treatment increased by 26%, 65%, and 101%, respectively, compared to co-culture control (Figure 7D). This result is consistent with our previous cell morphological analysis (Figure 5A) and viability assay results (Figure 5B).

### TPH104c significantly reduced TNBC intravasation from the primary tumor into the vasculature compared to PTX

**FIGURE 8:**
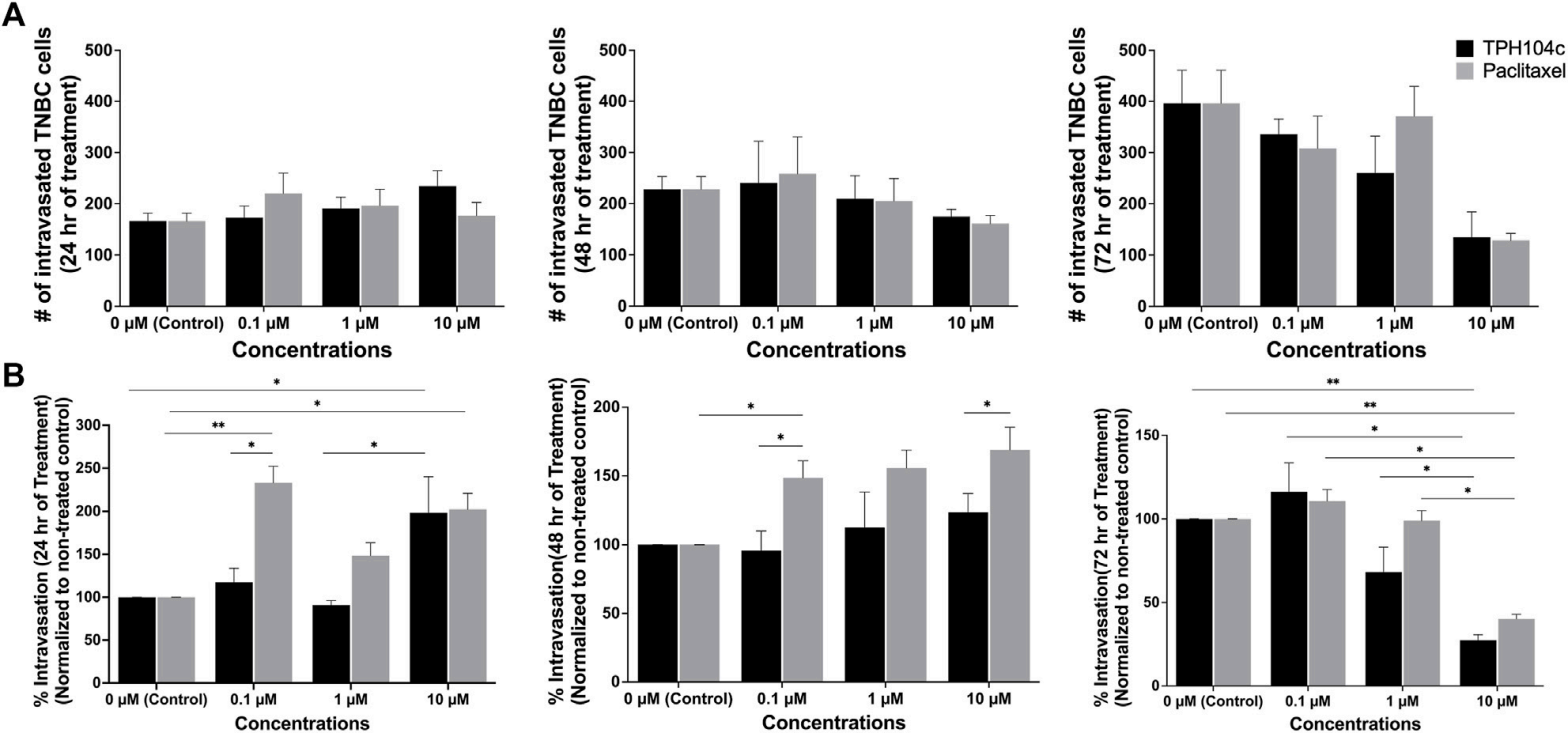
TPH104c significantly reduced the intravasation of TNBC cells from the primary tumor into the vasculature compared to PTX. Representative images of TNBC intravasation were shown in Figure 6A. **(A)** Quantification of the number of intravasated TNBC cells reveals that both treatments exhibit a concentration and time-dependent inhibitory effect. **(B)** By first normalizing the number of intravasated TNBC cells to the total number of TNBC cells in all the compartments, and then compare with control, we derive the “intravasation percentage” as a metric signifying TNBC intravasation. This index distinctly highlights TPH104c’s superior inhibitory effect in contrast to PTX. Interestingly, the intravasation percentage of TNBC cells increased after PTX treatment at 0.1 and 1 µM. Data are presented as mean ± SD (*n* = 3). *p < 0.05, **p < 0.01, by *ANOVA*.

Tumor cell intravasation is the process by which cancer cells escape from the primary tumor and enter the bloodstream (Sigdel et al., 2021). This allows cancer cells to travel to distant sites, forming metastases. Intravasation is a critical step in cancer progression and the formation of distant metastasis. Intravasation is regulated by complex interactions between tumor cells and the surrounding microenvironment, including the ECs forming blood vessel walls. Using CM-DiI fluorescent dye and time-lapse imaging, we monitored TNBC cells’ intravasation at various time points during the drug treatment in the tumor microenvironment. TNBC cell intravasation was first observed 48 h post-seeding (the day treatment starts). The number of intravasated cells increased steadily in the next 72 h (Figure 8A). A negative correlation was observed between drug treatment concentration and the number of intravasated TNBC cells.

The impact of drug treatments on TNBC intravasation is complicated by the decreased number of TNBC cells in the tissue compartment following drug treatment. To isolate the effect of drug treatments on TNBC intravasation, we analyzed the data by normalizing the number of intravasated cells to the number of cells remaining in the tissue compartment. This allowed us to obtain “intravasation percentage” data that revealed the portion of TNBC cells that were intravasated out of the total TNBC cell population at a given time. TPH104c treatment exhibited a concentration-dependent inhibition effect on TNBC intravasation (Figure 8B). In contrast, interestingly, the intravasation percentage of TNBC cells increased after PTX treatment at 0.1 or 1 µM. The exact mechanism for this increase is yet to be elucidated. However, it is reasonable to assume that the higher percentage of TNBC cells that were able to intravasate was due to the weakening of the endothelial barrier by PTX treatment, which reduces EC viability. At this point, significant intravasation into the vascular channels had occurred, as indicated by Figure 8B.

## Discussion

Microfluidic devices offer a promising tool for studying complex biological processes in a controlled environment (Sigdel et al., 2021). Compared to traditional compound and tissue testing, microfluidic testing can incorporate various levels of complexity that more closely mimic *in vivo* physiology, leading to more accurate results (Shang et al., 2019; Horowitz et al., 2020). Of greater interest, microfluidic devices can analyze drug transport and cancer metastasis (Ruzycka et al., 2019). The ability to recapitulate drug diffusion within a microfluidic device is valuable since it can simulate commonly observed delivery routes *in vivo* and is likely to represent real-world behavior. Furthermore, studying cancer metastasis is crucial since metastasis is one of the most significant factors in determining the outcomes of cancer treatment, especially for highly aggressive subtypes such as TNBC. Consequently, microfluidic approaches to analyzing BC metastasis are of considerable interest among researchers (Yankaskas et al., 2019; Sigdel et al., 2021).

In this study, we established a microfluidic tumor microenvironment model utilizing a commercially available platform which has the provision of co-culturing multiple cell types and providing a wide range of culturing conditions in one device due to its unique multi-compartment design. The device, fabricated in optically clear PDMS and bonded to a glass slide, comprises two semicircular vascular channels surrounding a tissue compartment interfaced via a porous architecture allowing for fluidic exchange, cell-cell interactions, and easy tracking of cell migration (Figure 1). The vascular channels and tissue compartment are individually perfused by precision syringe pumps via inlet/outlet ports connected via Tygon tubing. Utilizing this device, we successfully modeled the TNBC intravasation using human metastatic, drug-resistant TNBC cells (SUM159PTX) and human primary ECs (HUVEC) (Figure 3). HUVEC is a commonly used cell type in pre-clinical studies due to its availability, consistency, and extensive literature available for data comparison and validation (Lau et al., 2021). Its use in platform development allows for reliable replication of existing experimental models (Medina-Leyte et al., 2020). It is worth noting that ECs have been observed to respond to changes in shear levels in their *in vivo* environment (DeStefano et al., 2017). A dynamic perfusion scheme (0–1.75 dyne/cm2) was employed to facilitate EC physiological function and lumen formation (Figures 3, 4). This range of shear stresses is found in the tumor vasculature *in vivo* (Chary and Jain, 1989; Qazi et al., 2013; Stylianopoulos et al., 2018) and comparable to other reported microfluidic models (Buchanan et al., 2014; Jeon et al., 2015; Hynes et al., 2020; Saha et al., 2020; Azizgolshani et al., 2021; Ma et al., 2023).

The EC barrier plays a critical role in regulating the transport of molecules and cells between the blood and surrounding tissues. The integrity of the EC barrier limits the movement of substances through the intercellular spaces, including cancer cells. Disruption of the EC barrier can compromise its ability to prevent tumor cell intravasation, which can ultimately lead to the development of metastases in distant organs (Nguyen et al., 2009; Lou et al., 2016). Understanding the role of the EC barrier in the tumor microenvironment can inform the development of new strategies for cancer treatment and prevention. In our model, HUVEC established adherens junction in the vascular channel surface and aligned in the flow direction (Supplementary Figure S1), defining morphological features of the vascular homeostasis (Steward et al., 2015). The measured permeability of the EC barrier to 4 kDa dextran [(2.3567 ± 0.1002) × 10^–6^ cm/s] is comparable to observed microvessels permeability *in vivo* (Yuan et al., 2009; Shi et al., 2014) and is tighter than other reported *in vitro* models (Kazakoff et al., 1995; Lee et al., 2014; Frost et al., 2019). We choose fluorescent dextran in this range (4 kDa) for its comparable size to the two drug treatments (TPH104c: 390.47 Da and PTX: 853 Da). Compared to HUVEC, TNBC cells (SUM159PTX) were resuspended in 5% Matrigel and cultured in the tissue compartment without active flow perfusion. Instead, fresh media was given as a bolus injection every 8 h. The TNBC cells formed a 3D tumor 72 h after seeding, as confirmed by confocal imaging (Figure 4). TNBC co-culture negatively impacted barrier integrity (Supplementary Table S1; Figure 7), an observation that is consistent with our previous findings as the metastatic tumor can regulate VE-Cadherin and tight junction expression leading to increased EC permeability and cancer metastasis (Tang et al., 2017).

It is crucial to emphasize that the assessment of cytotoxicity to HUVECs in the well-plate culture does not necessarily reflect the cellular behavior within the microfluidic TME where both flow and TNBC cells may affect HUVEC viability and proliferation. This variation can be attributed to two key factors: 1) HUVECs’ proliferative state in static culture vs. under flow: HUVECs, like most endothelial cells, enter a quiescent state under appropriate flow stimulation (Doddaballapur et al., 2015; Roux et al., 2020; Ricard et al., 2021), remaining viable but not actively proliferating. This contrasts with their state in well-plate culture, where their doubling time spans 28–36 h (Lai et al., 2009; Lau et al., 2022) for early passage (<8) cells. This difference significantly impacts their response to TPH104c treatment, as TPH104c targets mitochondrial fission—a process more pronounced in actively dividing cells compared to quiescent ones (Malla et al., 2022a). 2) The impact of TNBC on HUVEC viability and function: Our permeability data (Supplementary Table S1; Figure 7) indicate that the presence of TNBC co-culture adversely affected EC barrier integrity even in the absence of drug treatment. This influence of TNBC on ECs aligns with our prior study (Tang et al., 2017) and corroborates findings in existing literature (Mierke, 2011; Mierke, 2012). Moreover, a significant increase in EC permeability, indicating a more permeable EC barrier, was evident following drug treatment, when comparing the EC-only group and the TNBC co-culture group (Supplementary Table S1). The precise underlying mechanism for this variance remains incompletely understood; however, it is widely postulated that the excessive cytokine release within the TME following apoptosis-inducing chemotherapy plays a pivotal role. In summary, the disparities observed in cytotoxicity assessments between the well plate culture and the co-culture system underscore the importance of context-specific evaluations. These insights not only enhance our understanding of cellular responses but also underscore the intricate nature of therapeutic assessments within diverse microenvironments.

The intravenous route of drug delivery was successfully modeled using two chemotherapeutics, PTX and TPH104c. The two drug treatments, introduced via the vascular channels, successfully reached the TNBC tumor grown in tissue compartment, resulting in both time and concentration dependent TNBC tumor growth inhibition (Figures 5, 6). PTX is one of the most active and widely used chemotherapeutics agents in BC treatment (Awada et al., 2014). However, its adverse effects on healthy cells, especially vascular ECs(Pasquier et al., 2004; Pasquier et al., 2005; Bocci et al., 2013), have limited its application. In our model, PTX treatment significantly reduced EC barrier integrity as evident by cell viability assay (Figure 5) and the permeability assay (Figure 7). The underlying mechanism of PTX’s endothelial toxicity is not fully understood, but it is believed to involve disruption of microtubule dynamics (Pasquier et al., 2005; Bocci et al., 2013), a crucial structure that helps maintain cell shape and enable movement of various cellular components. ECs are particularly sensitive to changes in microtubule dynamics, which are required for shear-induced changes in EC shape and planar cell polarity (McCue et al., 2006). Upon disruption of these structures by PTX treatment, ECs can no longer maintain their tight monolayer structure under flow shear, leading to increased TNBC intravasation upon PTX treatment (Figure 8). Our result is consistent with previous studies in the literature, which have reported similar effects of PTX (Volk-Draper et al., 2014; Ren et al., 2015; Karagiannis et al., 2017b; Chang et al., 2017), indicating PTX induced EC damage may contribute to chemotherapy exacerbated cancer metastasis.

Compared to PTX, the novel thieno-pyrimidin-4-yl-hydrazinylidene analog TPH104c exhibited significantly reduced EC toxicity (Figures 5, 7). As TPH104c largely preserved EC barrier function, the intravasation percentage of TNBC cells dropped significantly upon TPH104c treatment (Figure 8) compared to PTX treatment, although their anti-TNBC efficacy were similar (Figures 5, 6). The selectivity of TPH104c for cancer cells over ECs may be attributed to the difference in the degree of dynamin-related protein 1 (Drp1) activity between normal and cancer cells (Zhao et al., 2013; Chen and Chan, 2017; Lima et al., 2018; Grasso et al., 2020). TPH104c induces non-apoptotic cell death, which is regulated by Drp1, an essential protein for mitochondrial and peroxisomal fission (Malla et al., 2022a). The cytotoxic potential of TPH104c is dependent on the amount of Drp1 present in cells. Therefore, selective inhibition of TNBC growth can be achieved because Drp1 activity is significantly higher in TNBC cells than normal cells (Zhao et al., 2013; Chen and Chan, 2017; Lima et al., 2018; Grasso et al., 2020). The main goal of the current study is to establish the feasibility of the microfluidic tumor microenvironment model for simulating drug transport and tumor cell intravasation. Nonetheless, it is feasible to construct even more intricate models based on the existing framework to delve into fundamental aspects of the tumor microenvironment. These extended models could explore intricate dynamics, including the impact of TNBC on EC barrier characteristics and the interactions between tumors and immune cells. Particularly noteworthy is the potential to investigate the influence of the TPH compounds in fostering an immunogenic tumor microenvironment (Tukaramrao et al., 2021). Such models can provide direct evidence of immune cell recruitment and maturation upon TPH compound treatment and allow us to gain mechanistic insights into how different cell death mechanisms can change anti-tumor immunity.

## Conclusion

We developed an *in vitro* microfluidic model of TNBC microenvironment utilizing an existing commercial platform. Overall, this model presents an innovative approach to anti-metastatic efficacy testing by incorporating co-cultured human TNBC cells and primary ECs within an optically clear device, reproducing tumor perfusion and allowing for real-time assessment of therapeutic responses and interactions between the tumor and ECs, thus addressing a critical need for high-fidelity *in vitro* testing of anticancer therapeutics. Using this model, we demonstrat the selectivity of the novel compound TPH104c towards TNBC cells over ECs, replicating systemic drug delivery. Our findings indicate that chemotherapy-induced EC damage may promote cancer metastasis while preserving EC barrier integrity may reduce cancer cell intravasation. Overall, this microfluidic model enables the study of anti-metastatic therapies using human cells under tunable conditions. This platform has the potential to facilitate the rapid development of biomimetic systems to identify molecular targets and screen promising therapeutics against cancer metastasis.

## Data Availability

The raw data supporting the conclusion of this article will be made available by the authors, without undue reservation.

## Abbreviations

3D, Three Dimensional; *ANOVA*, Analysis of Variance; BC, Breast Cancer; BF, Bright Field; Da, Dalton; DI water, Deionized water; DMEM, Dulbecco’s Modified Eagle Medium; DPBS, Dulbecco’s Phosphate Buffered Saline; EC, Endothelial Cell; EGM, Endothelial Cell Growth Medium; hr, hour; IHC, Immunohistochemistry; min, minute; MTT, (3-(4,5-Dimethylthiazol-2-yl)-2,5-diphenyltetrazolium bromide); PBS, Phosphate Buffered Saline; PDMS, Polydimethylsiloxane; psi, pounds per square inch; PTX, Paclitaxel; ROI, Region of Interest; sec, second; SD, Standard deviation; TME, Tumor microenvironment; TNBC, Triple-Negative Breast Cancer; TPH104c, Thienopyrimidine Analog.
