# Supplementary material for: Biomimetic on-chip assay reveals the anti-metastatic potential of a novel thienopyrimidine compound in triple-negative breast cancer cell lines
Source: Front Bioeng Biotechnol. 2023 Sep 28;11:1227119. doi: 10.3389/fbioe.2023.1227119 (PMC10569307; doi:10.3389/fbioe.2023.1227119)
Supplement: Supplementary file 1 [file DataSheet1.pdf]

Supplementary Material

Biomimetic on-chip assay reveals the anti-metastatic potential of a novel thienopyrimidine compound in triple-negative breast cancer cell lines

Indira Sigdel[1], Awurama Ofori-Kwafo[1], Robert Joseph Heizelman III[2], Andrea Nestor-Kalinoski[3], Balabhaskar Prabhakarpandian[4], Amit K. Tiwari[5], Yuan Tang[1, *]

[1]Department of Bioengineering, College of Engineering, University of Toledo, Toledo, OH, USA

[2]Department of Biomedical Engineering, College of Engineering, University of Michigan, Ann Arbor, MI, USA

[3]Department of Surgery, College of Medicine & Life Sciences, University of Toledo, Toledo, OH, USA

[4]Biomedical Technology, CFD Research Corporation, Huntsville, AL, USA

[5]Department of Pharmacology and Experimental Therapeutics, College of Pharmacy & Pharmaceutical Sciences, University of Toledo, Toledo, OH, USA

*** Correspondence:**

Yuan Tang, PhD

yuan.tang@utoledo.edu

Total Number of Pages: 9

Number of Figures: 6

1. Steps for counting cells using the "Count and Measure" Function in cellSens Dimension software

1) Open the Image that you want to know how many TNBCs are present in the channels.
2) Use **View> Tool Windows > Count and Measure** command to have your Count and Measure window displayed in the software.
3) Open the **Options** dialog box by clicking on **Count and Measure** options button.
4) Select **Detection** entry.
5) In the **Options** group, set the **Minimum object size** to 5.
6) Click **Ok.**
7) In **Count and Measure** tool window, select **Automatic Threshold** option in the **Threshold** menu.
8) All objects that have been detected are displayed in color.
9) Make sure all objects to be counted are selected.
10) If objects are not correctly recognized: Go to **Background > Dark** option.
11) Delete phases from earlier analysis by clicking **Remove Phase** button until the button becomes inactive.
12) Click **Count and Measure** button in **Automatic Threshold** dialog box.
13) The numbers of objects detected will be shown in the **Count and Measure** tool Window in the **Object Count** group.

Table S1. Measured permeability values (n=3, mean ± SD)

Treatment Groups	Permeability (cm/s)
Empty Channels	$(2.07 \pm 0.104) \times 10^{-5}$
**EC only group**	
No treatment (0 μm)	$(1.05 \pm 0.020) \times 10^{-6}$
10 μm TPH104c	$(2.65 \pm 0.161) \times 10^{-6}$
10 μm Paclitaxel	$(3.91 \pm 0.321) \times 10^{-6}$
**Co-culture group**	
No treatment (0 μm)	$(2.357 \pm 0.100) \times 10^{-6}$
0.1 μm TPH104c	$(2.358 \pm 0.388) \times 10^{-6}$
0.1 μm Paclitaxel	$(2.812 \pm 0.873) \times 10^{-6}$
1 μm TPH104c	$(3.070 \pm 0.279) \times 10^{-6}$
1 μm Paclitaxel	$(3.758 \pm 0.638) \times 10^{-6}$
10 μm TPH104c	$(3.672 \pm 0.077) \times 10^{-6}$
10 μm Paclitaxel	$(4.763 \pm 0.259) \times 10^{-6}$

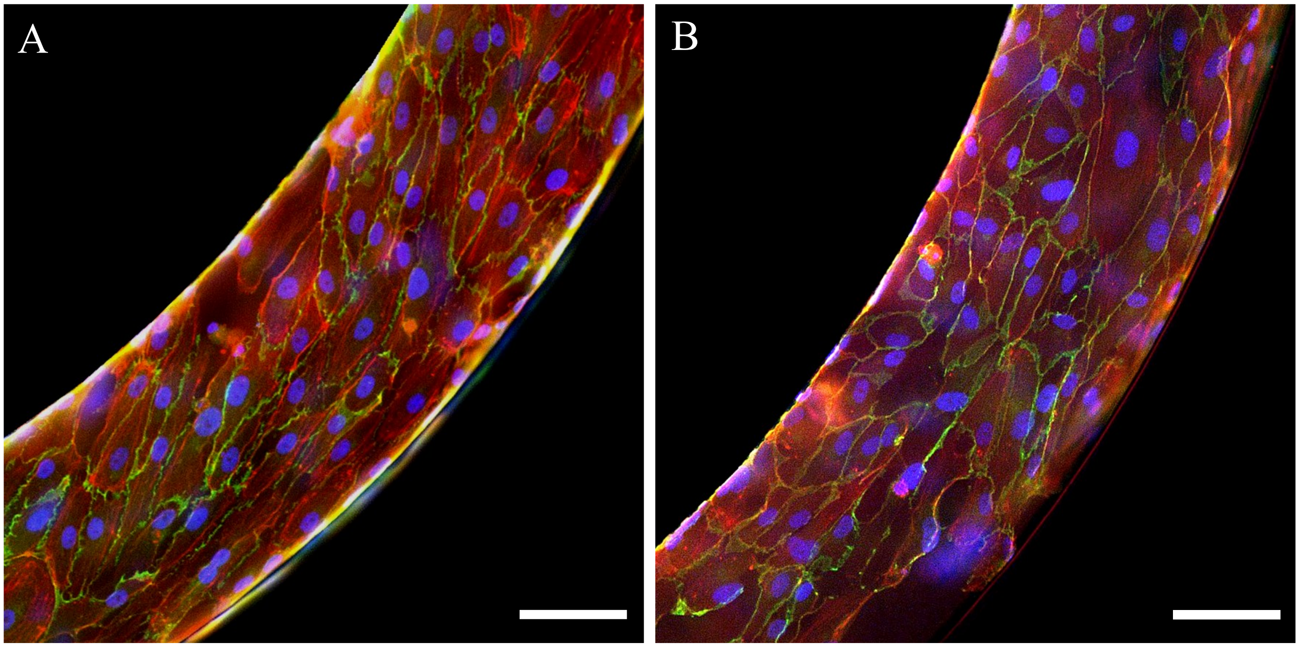

Figure S1. Dynamic vs. static culture of HUVECs inside the vascular channels of the microfluidic device. (**A**) Under continuous flow perfusion (0-1.75 dyne/cm^2) HUVECs express strong intercellular contact as indicated by a prominent, continuous adherent junction signal (VE-cadherin) and well-aligned stress fibers (Phalloidin) towards flow direction. (**B**) Without flow shear stimulation, stress fibers expression is significantly downregulated and disoriented; VE-Cadherin expression is inhomogeneous and sporadic. Images were taken after 48 hr of flow perfusion (**A**) or static culture (**B**). Red: TNBC cells (CM-DiI), Blue: Cell nucleus (Hoechst 33342), and Green: adherens junction between HUVECs (VE-Cadherin). Scale bar = 100 μm.

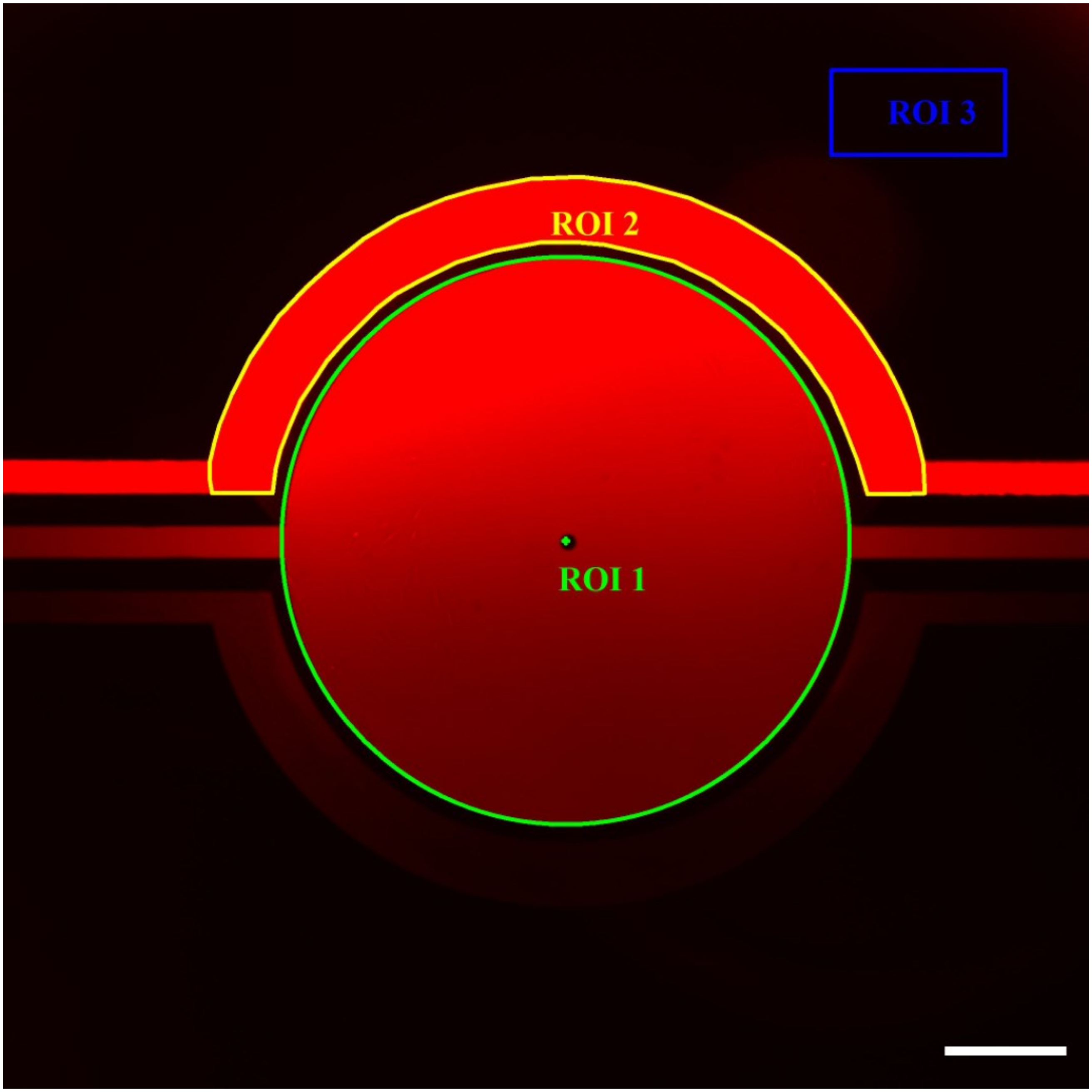

Figure S2. ROIs were defined for permeability calculation. Intensity values measured in ROIs 1-3 represent tissue compartment during dextran perfusion, vascular channels during dextran perfusion, and the area outside the experimental region (background), respectively. ROI 3 was subtracted from ROI 1 and ROI 2 when calculating the permeability value. Scale bar = 400 µm.

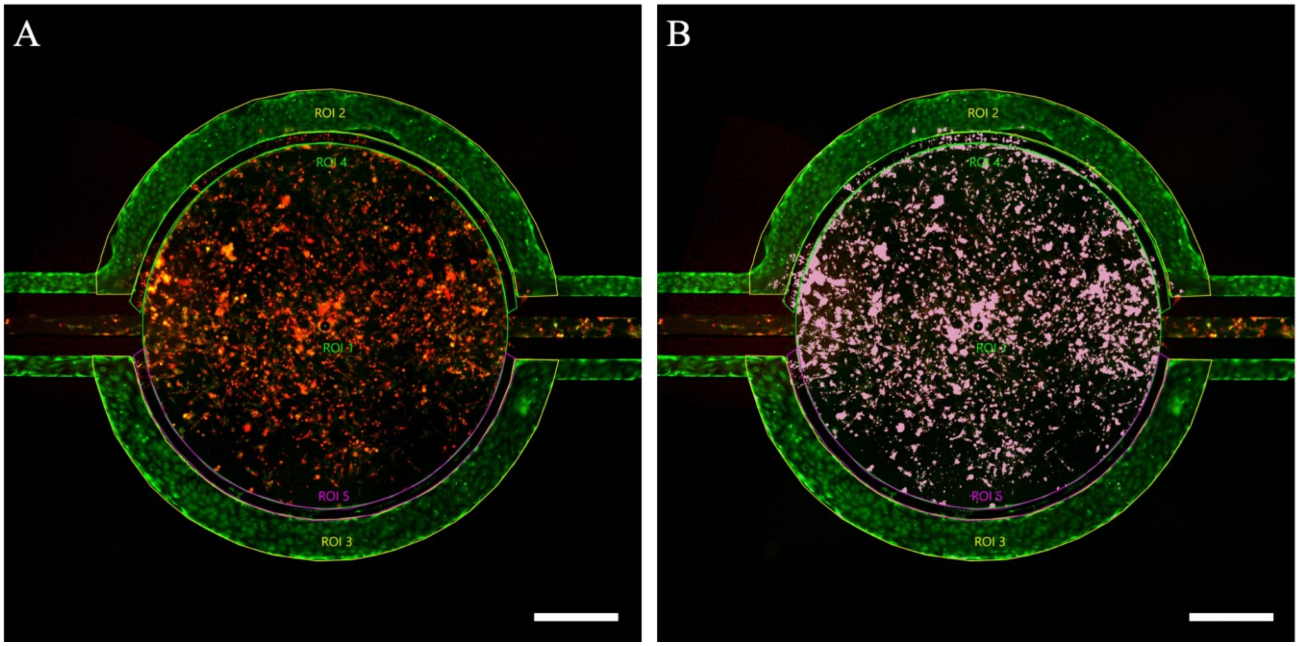

Figure S3. A representative merged image of the microfluidic TME before (A) and after (B) applying "Count and Measure" function. TNBC cells were stained with CM-DiI (red). HUVECs were stained with CMFDA (green). The number of TNBC cells were counted in ROIs 1-5 to facilitate intravasation quantification. ROI 1: tissue compartment; ROIs 2-3: vascular channels; ROIs 4-5: porous interfaces. Percentage intravasation were calculated based on (ROI 2 + ROI 3) / (ROI 1 + ROI 2 + ROI 3 + ROI 4 + ROI 5). Scale bar = 400 µm.

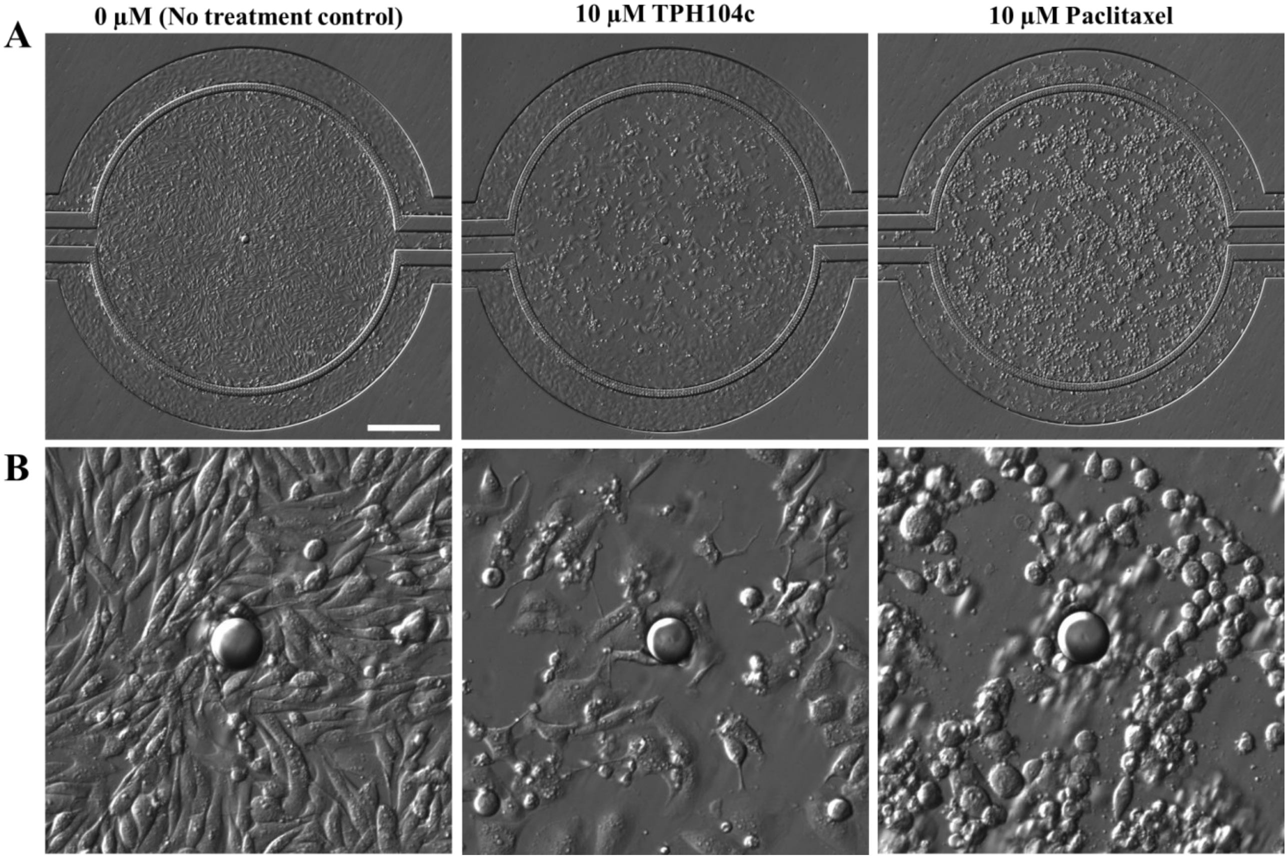

Figure S4. (A) Representative Brightfield images (BF) showing morphological changes in TNBC cells (10X magnification) after treatment with EGM only (non-treated control, 0 μM), 10 μM TPH104c or 10 μM PTX respectively for 72 hr. (B) Zoomed images of TNBC cells in tissue compartment after 72 hr of treatment. Scale bar = 400 μm.

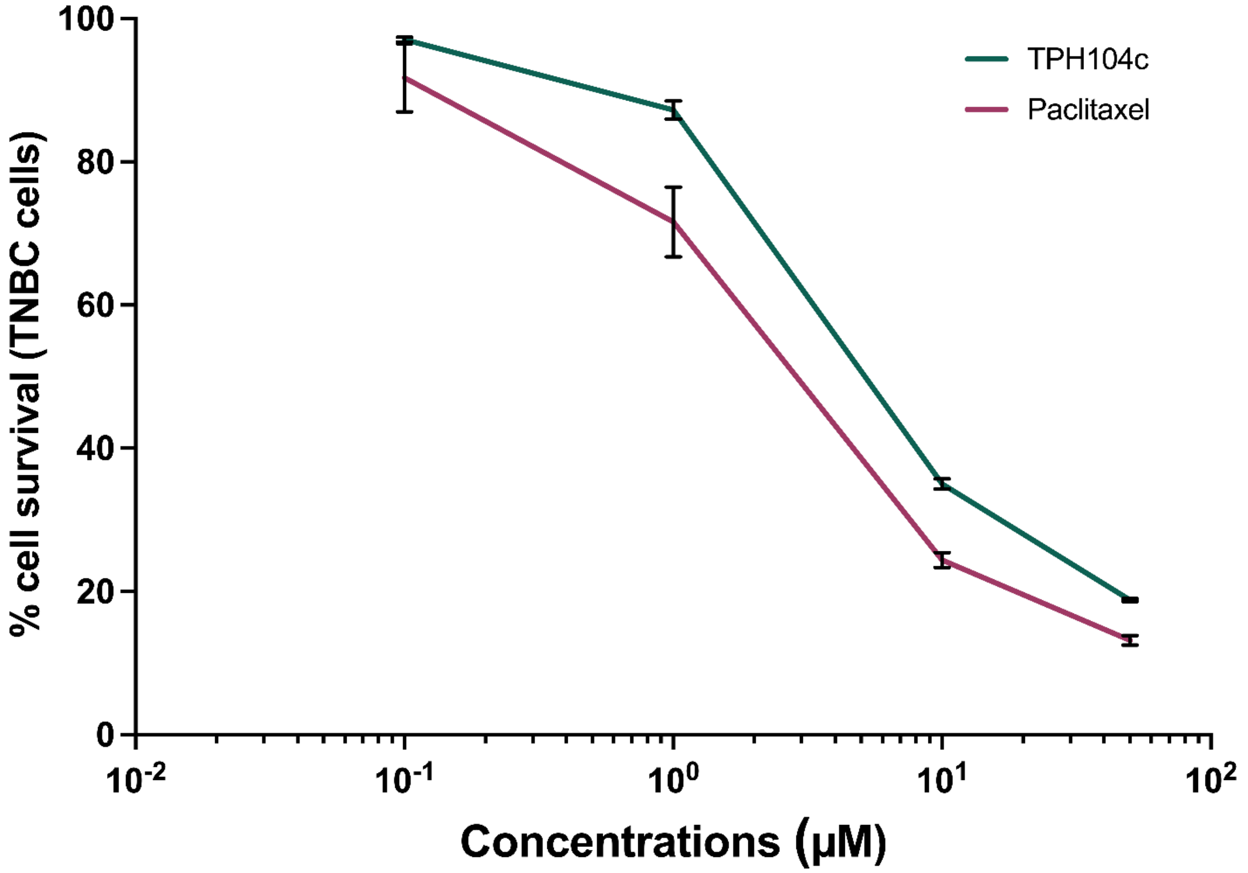

Figure S5. Dose-response curve of TNBC cells treated with various concentrations of TPH104c and PTX in 96-well plates (n=3). Cell survival after each drug treatment was determined by MTT assay. The calculated IC_{50} values for TPH104c and PTX treatment are 7.41 µM and 5.12 µM respectively.

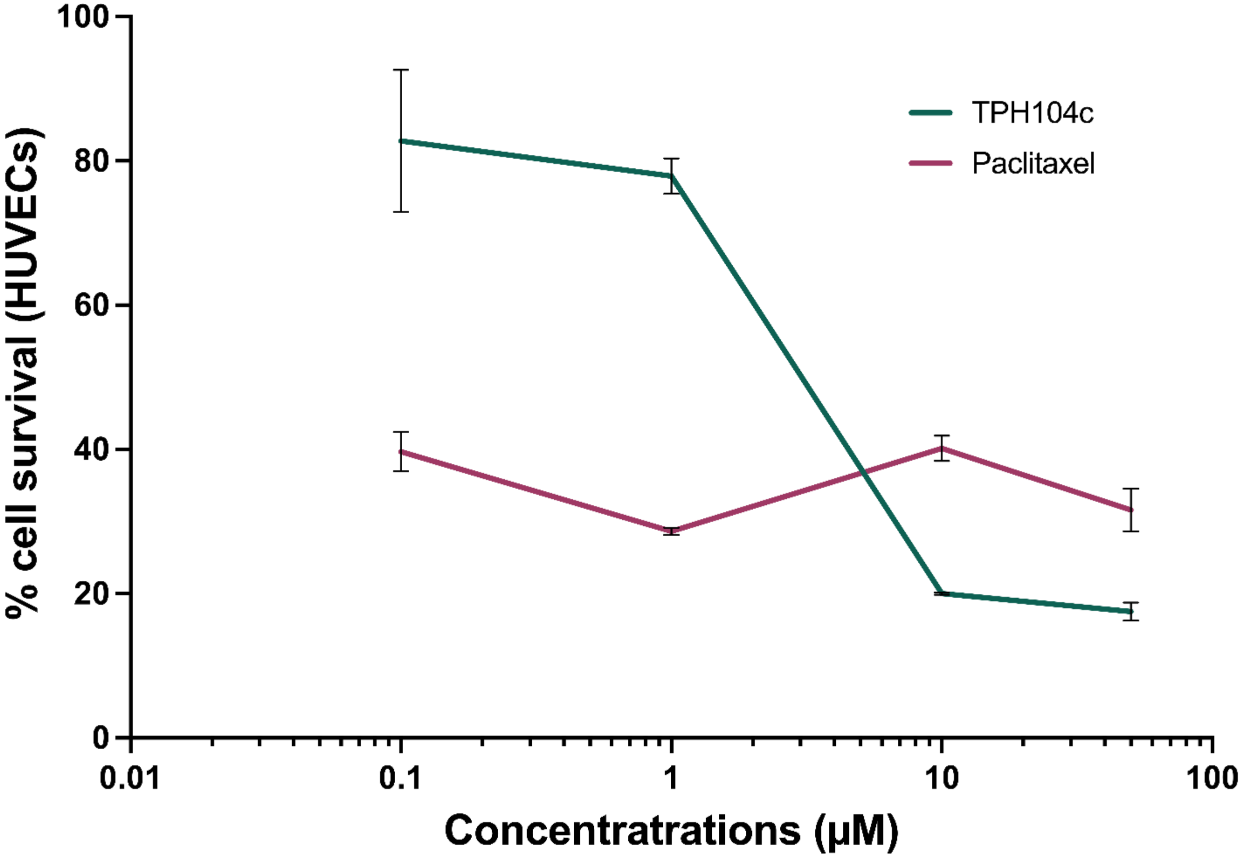

Figure S6. Dose-response curve of HUVECs treated with various concentrations of TPH104c and PTX in 96-well plates (n=3). Cell survival after each drug treatment was determined by MTT assay. The calculated IC_{50} values for TPH104c and PTX treatment are 5.34 µM and 0.075 µM respectively.
